# Pre-exposure Prophylaxis Outcomes in High-Risk Individuals in Saudi Arabia: A 48-Week Observational Cohort Study

**DOI:** 10.7759/cureus.111259

**Published:** 2026-06-21

**Authors:** Ali Alsaeed

**Affiliations:** 1 Infectious Disease Division, Department of Internal Medicine, Dammam Medical Complex, Dammam, SAU

**Keywords:** human immunodeficiency virus, middle east and north africa, preexposure prophylaxis, prep adherence, retention in care, saudi arabia, seroconversion, tenofovir alafenamide/emtricitabine

## Abstract

Background

Human immunodeficiency virus (HIV) continues to pose a substantial global health challenge, with an increasing burden observed in the Middle East and North Africa (MENA) region despite advancements in preventive measures. Pre-exposure prophylaxis (PrEP) has demonstrated high efficacy globally; however, data from real-world settings within the MENA region, particularly Saudi Arabia, remain limited. This study aims to evaluate the effectiveness, safety, adherence, and implementation outcomes of PrEP among a high-risk population.

Methods

This prospective, single-center, observational cohort study was conducted at Dammam Medical Complex, Saudi Arabia. A total of 50 HIV-negative adults at high risk of HIV infection were enrolled and followed for 48 weeks. Participants received tenofovir alafenamide/emtricitabine (TAF/FTC) as either daily or on-demand PrEP. The primary outcome was HIV incidence per 100 person-years. Secondary outcomes included retention in care, visit adherence, safety (renal, hepatic, and bone), adherence levels, sexually transmitted infection (STI) incidence, and implementation challenges. Descriptive statistics and paired analyses were performed.

Results

Human immunodeficiency virus seroconversions were not observed over 48 weeks, yielding an incidence of 0 per 100 person-years (95% CI: 0.0-0.071). Retention in care was high (94%), with similar visit adherence (94%). Adherence exceeded 90% in 92% of participants, with 100% adherence among daily PrEP users. Adverse events were infrequent (6%) and mild, with no discontinuations due to safety concerns. Small but statistically significant changes in renal function and bone mineral density were observed, without clinical relevance. STI incidence was 35.4 per 100 person-years, with 30% of participants acquiring at least one STI. High-risk behaviors persisted, including condomless sex (68%).

Conclusion

Tenofovir alafenamide/emtricitabine-based PrEP demonstrated excellent effectiveness, safety, and adherence in a real-world setting in Saudi Arabia. Despite these favorable outcomes, high STI incidence and ongoing risk behaviors highlight the need for integrated prevention strategies. These findings support the expansion of PrEP programs in MENA while emphasizing the importance of comprehensive sexual health interventions and larger multicenter studies.

## Introduction

Human immunodeficiency virus (HIV) was initially identified in the early 1980s and continues to pose a substantial challenge to global health, having resulted in approximately 44.1 million fatalities to date [1, 2]. By the end of 2024, it was estimated that approximately 40.8 million individuals worldwide were living with HIV, with 65% residing in the World Health Organization (WHO) African Region. In that year, there were 1.3 million new infections and 6,30,000 deaths related to HIV [2, 3]. The primary modes of transmission for HIV include sexual contact and vertical transmission from mother to child during childbirth or breastfeeding [1]. The Middle East and North Africa (MENA) region constitutes approximately 10% of the global population [4]. Since 2015, there has been a 24% increase in new HIV infections and a 12% rise in AIDS-related fatalities, with an estimated 4,20,000 individuals living with HIV, including 14,000 children [5]. According to the Joint United Nations Programme on HIV/AIDS (UNAIDS), there has been a 116% increase in new infections from 2010 to 2023. In 2022, key populations and their sexual partners accounted for an estimated 84% of new HIV infections, with particularly sharp increases observed among gay men and other men who have sex with men (MSM) [6]. Despite the growing burden, substantial gaps still persist in diagnosis and treatment access [4, 5].

Pre-exposure prophylaxis (PrEP), predominantly utilizing oral tenofovir disoproxil fumarate/emtricitabine (TDF/FTC), has emerged as a cornerstone in HIV prevention strategies, offering highly effective protection for individuals at substantial risk of HIV infection [7, 8]. The iPrEx trial included 2,499 HIV-seronegative men or transgender women (TGW) who have sex with men and were randomly assigned to receive a once-daily oral combination of FTC and TDF or placebo. Participants were followed for a total of 3,324 person-years (median follow-up: 1.2 years; maximum: 2.8 years). At enrollment, 10 individuals were already infected with HIV, while 100 new infections occurred during follow-up (36 in the TDF-FTC group and 64 in the placebo group). This corresponded to a 44% reduction in HIV incidence among those receiving TDF-FTC (95% CI: 15-63; P = 0.005) [9, 10].

Further, the Partners PrEP study, conducted on a total of 4747 couples (1584 assigned to TDF, 1579 to TDF-FTC, and 1584 to placebo), found that 62% of the couples had an HIV-1-seronegative partner who was male. During follow-up, 82 HIV-1 infections occurred among seronegative participants: 17 in the TDF group (incidence 0.65 per 100 person-years), 13 in the TDF-FTC group (0.50 per 100 person-years), and 52 in the placebo group (1.99 per 100 person-years). This corresponded to a 67% reduction in HIV-1 incidence with TDF (95% CI, 44-81; P<0.001) and a 75% reduction with TDF-FTC (95% CI, 55-87; P<0.001) [11]. Another observational study of participants from ANRS IPERGAY trial among Men and TGW who have sex with men (n=361) on-demand TDF (300 mg) and FTC (200 mg) reported HIV incidence of 0·19 per 100 person-years (95% CI: 0·01-1·08), compared with 6·60 per 100 person-years (95% CI: 3·60-11·05) in the placebo group indicating a relative reduction of 97% (95% CI: 81-100) in the incidence of HIV with on demand PrEP [12].

In the MENA region, efforts to control HIV face serious challenges. A systematic review and meta-analysis including 228,403 MSM participants reported that 58.6% (95% CI: 54.8-62.4) of them showed willingness to use PrEP [13]. However, actual utilization remains low due to stigma, cost, side‑effect concerns, and limited awareness [14, 15]. As a result, this region is one of the few in the world where HIV cases are still rising quickly, which makes it a major concern in the global fight against the epidemic. Treatment access is also limited: only about 43% of people living with HIV in MENA receive antiretroviral therapy, the lowest coverage rate of any region worldwide [16]. Young adults are disproportionately impacted by the HIV epidemic in MENA, with nearly one-fifth of new infections occurring among those aged 15-24 years [6]. Although PrEP has demonstrated strong efficacy in preventing HIV globally, there is a lack of clinical studies from the MENA region [5, 14].

Clinical studies have shown that monitoring renal and bone health is essential for individuals using tenofovir-based PrEP regimens [17, 18]. Adherence has been consistently identified as the critical determinant of PrEP effectiveness. PrEP adherence is multifaceted and needs to be understood in relation to fluctuating HIV risk and the concurrent use of other prevention strategies [19]. However, in the MENA region, HIV is severely understudied despite the growth in new infections since 2010 [20]. Most countries lack national sexually transmitted infection (STI) screening programs, and the available data are largely derived from symptomatic individuals. This gap in surveillance makes it difficult to capture the true burden of STIs, particularly in Saudi Arabia. Establishing robust surveillance systems, addressing stigma and barriers to healthcare access, and expanding STI screening and vaccination programs are therefore essential to inform HIV prevention strategies [15].

Taken together, these challenges underscore the urgent need for locally generated evidence on PrEP outcomes to guide policy and implementation in the MENA region. Moreover, data on safety monitoring, retention in care, sexually transmitted infection dynamics, and the feasibility of different dosing strategies in routine clinical practice are scarce. Addressing these gaps is essential to inform contextually relevant HIV prevention strategies and optimize PrEP implementation in high-risk populations. Therefore, the primary objective of this study was to evaluate HIV incidence over 48 weeks among high-risk individuals receiving PrEP, while also assessing retention and visit adherence, monitoring safety outcomes (renal, bone mineral density (BMD), hepatic), determining STI incidence, analyzing adherence by dosing regimen, and identifying implementation challenges in the Saudi Arabian context.

## Materials and methods

Study design and setting

This was a prospective, single-center, observational cohort study conducted at Dammam Medical Complex, Saudi Arabia. The study evaluated real-world outcomes of PrEP use over a 48-week follow-up period from June 2024 to May 2025, among individuals at high risk of HIV infection. The study was designed to reflect routine clinical practice, including patient-driven selection of PrEP dosing strategies and standard-of-care monitoring.

Study population

A total of 50 HIV-negative individuals at significant risk of HIV infection enrolled in the PrEP program. Eligible participants were adults aged 18 or older exhibiting high-risk behaviors, such as MSM, individuals with multiple sexual partners, those engaging in sex without condoms, and HIV-negative partners of people living with HIV. Participants were mandated to possess a verified negative HIV test result at the outset and demonstrate willingness to commence PrEP, along with adherence to scheduled follow-up visits.

Sample size estimation

A sample size of 50 participants was determined to be sufficient to evaluate the effectiveness of a 48-week PrEP program in preventing HIV acquisition among high-risk HIV-negative individuals. The primary endpoint is the HIV seroconversion rate during the 48-week follow-up period, expressed as the incidence of seroconversion per 100 person-years.

The sample size was calculated using standard estimation methods for proportions, assuming a confidence level of 90% and a margin of error of ±5%. A conservative expected population proportion (seroconversion rate) of 5% was used, with a finite population size of 1,000 individuals. Based on these assumptions, a minimum of 50 participants was required to estimate the HIV seroconversion rate with acceptable precision, ensuring that the observed incidence will be within 5 percentage points of the true population value with 90% confidence.

Therefore, enrolling at least 50 high-risk HIV-negative individuals and following them over the 48-week period was estimated to provide sufficient statistical precision to assess the effectiveness of the PrEP program in reducing HIV acquisition.

The size calculation for a single proportion is as follows [21]:

$n_0 = \dfrac{Z^2 \cdot p \cdot (1 - p)}{d^2}$

where Z^2^ is the Z value for the confidence level, p is the expected proportion, and d is the margin of error.

For a finite population, the corrected sample size is:

$n = \dfrac{n_0}{1 + \dfrac{n_0 - 1}{N}}$

The confidence level = 90%, so Z=1.645; the expected seroconversion proportion p=0.05; the margin of error d=0.05; the finite population N=1000; the size of the population = n; and Cochran’s sample size is computed using the formula for ideal sample size = n_0_.

Inclusion and exclusion criteria

Participants were eligible if they were aged 18 or older, confirmed HIV-negative at the start, identified as being at significant risk of HIV due to behavioral or partner-related factors, and willing to start PrEP with adherence to scheduled follow-up and monitoring.

Participants were excluded if they had confirmed or suspected HIV infection at baseline, significant renal impairment (estimated glomerular filtration rate (eGFR) <60 mL/min/1.73 m²), known hypersensitivity or contraindication to tenofovir alafenamide or emtricitabine, active severe hepatic disease, clinically significant baseline laboratory abnormalities, or if they could not or were unwilling to provide informed consent or follow study procedures.

Intervention and dosing strategy

The study utilized a real-world, evidence-based observational design while incorporating a structured clinician-directed approach for the initiation of HIV PrEP among individuals identified as being at high risk for HIV acquisition. All eligible participants were initiated directly on the standard recommended PrEP regimen comprising tenofovir alafenamide 25 mg/emtricitabine 200 mg (TAF/FTC), without dose titration or gradual escalation, in accordance with contemporary international clinical practice guidelines, including those of the Centers for Disease Control and Prevention and the WHO.

Participants receiving the daily oral PrEP regimen (n=10, 20%) were prescribed TAF/FTC as one tablet administered orally once daily at approximately the same time each day, with or without food. This regimen was preferentially recommended for individuals with persistent or ongoing HIV exposure risk, including those with an HIV-positive partner with unknown or detectable viral load, as well as individuals reporting regular condomless sexual intercourse.

Participants assigned to the event-driven (on-demand) PrEP regimen (n=40, 80%) received TAF/FTC according to the 2-1-1 dosing strategy, consisting of two tablets administered orally between 2 and 24 hours prior to anticipated sexual exposure, followed by one tablet 24 hours after the initial dose and a final tablet 48 hours after the initial dose. This regimen was offered exclusively to cisgender men who were able to reliably anticipate sexual activity, consistent with evidence-based recommendations supporting the use of on-demand PrEP within this population.

Selection of the PrEP regimen was individualized and determined jointly by the treating clinician and participant at the time of enrollment, based on assessment of sexual behavior patterns, HIV risk profile, lifestyle considerations, and participant preference. No dose adjustments, titration schedules, or escalation protocols were implemented in either treatment arm. In addition, all participants received standardized counseling at baseline and during each follow-up visit regarding appropriate dosing schedules, adherence strategies, the importance of consistent PrEP use, and management of missed doses.

Follow-up and data collection

Participants were monitored for up to 48 weeks, averaging about 46 weeks. They attended scheduled visits starting with a baseline assessment, followed by regular follow-ups for clinical evaluations, adherence checks, and laboratory tests. Data gathered included demographic details, initial risk behaviors, prior STI history, PrEP regimen choices, adherence patterns, and clinical outcomes.

Outcome measures

Primary Outcome

The incidence of HIV seroconversion over the 48-week follow-up period, expressed as cases per 100 person-years.

Secondary Outcomes

The secondary outcomes included retention in care, defined as the proportion of participants who complete the 48-week follow-up period, and visit adherence, assessed through attendance at scheduled follow-up visits. Safety outcomes were evaluated by monitoring renal function using serum creatinine and eGFR, BMD at the lumbar spine and hip through T-scores, hepatic parameters such as alanine transaminase levels, and the occurrence of adverse events (AEs). Adherence was assessed based on self-reported adherence levels, with comparisons drawn between daily and on-demand regimens. Additionally, the incidence of sexually transmitted infections (STIs), including both the number and types of new infections during the follow-up period, was recorded. The study also explores implementation challenges, including medication supply interruptions and other related barriers.

Laboratory assessments

All participants received extensive laboratory testing both at baseline and during follow-up, which included HIV testing, assessments of renal and liver function, BMD scans, STI screening, hepatitis B and C tests, and complete blood counts.

Adherence assessment

Adherence was evaluated through self-reporting and classified into categories: >90%, 80-90%, or <80%. Data on barriers to adherence, such as interruptions in medication supply, were also collected. Adherence counseling was offered at every visit.

Management of STIs and risk behavior assessment

Participants routinely underwent STI screening and received treatment following standard clinical guidelines. When appropriate, partner notification and repeat testing were performed. Self-reported data on risk behaviors, such as condom use and the number of sexual partners, were collected.

Statistical analysis

Descriptive statistics summarized baseline characteristics and study outcomes. Continuous variables are presented as mean ± standard deviation, while categorical variables are shown as frequencies and percentages. HIV incidence was calculated per 100 person-years, along with 95% confidence intervals. Paired statistical tests compared baseline and week 48 parameters, with p-values less than 0.05 indicating statistical significance.

Ethical considerations

The study was conducted in accordance with the Declaration of Helsinki. Institutional ethical approval was obtained prior to study initiation (Dammam Medical Complex Institutional Review Board issued approval IM-62), and all participants provided written informed consent. Participant confidentiality was maintained throughout the study.

## Results

A total of 50 participants were enrolled in the PrEP program at Dammam Medical Complex. Of the enrolled cohort, 45 were male and five were female, with a median age of 30 years (range 18-50). Among these, five female participants were the HIV-negative partners of people living with HIV, while male participants reported multiple sexual partners and other high-risk behaviors. At baseline, 60% of the cohort had a documented history of at least one STI (most commonly gonorrhea or syphilis), and 24% had an HIV-positive primary partner. Forty (80%) participants preferred the on-demand PrEP dosing protocol, and 10 (20%) opted for daily PrEP. All 50 participants were followed for a mean duration of 46.08 ± 7.80 weeks.

HIV incidence, retention in care, and visit adherence

No HIV seroconversions occurred during the 48-week follow-up period. The incidence rate was calculated as 0 per 100 person-years (95% CI: 0.0 to 0.071), indicating excellent preventive effectiveness of PrEP throughout the study. Participant retention was high, with 47 individuals (94%) completing the 48-week follow-up. Two participants (4%) were lost to follow-up, and one (2%) discontinued for reasons unrelated to AEs. No discontinuations were due to AEs. Visit adherence was also strong: 47 participants (94%) attended all scheduled visits, while three (6%) missed two or more visits. Refer to Table 1 for additional details.

**Table 1:** HIV incidence and retention in care and adherence outcomes over 48 weeks Incidence rates are reported per 100 person-years, with 95% confidence intervals (CIs). Continuous variables appear as mean ± standard deviation (SD). Retention in care covers 48-week completion status and discontinuation reasons. Visit adherence measures attendance at scheduled follow-ups. Descriptive outcomes do not include statistical tests; p < 0.05 indicates significance. AEs, Adverse events; SD, Standard deviation

Outcome Measure	Result	Percentage (%)	Statistical Significance	
Number of HIV seroconversions	0	0	–	
Retention in Care	-	-		
Completed 48-week follow-up	47	94.0	–	
Lost to follow-up	2	4.0	–	
Discontinued due to AEs	0	0	–	
Discontinued due to other reasons	1	2.0	–	
Visit Adherence				
Attended all scheduled visits	47	94.0	–	
Missed two or more visits	3	6.0	–	
Incidence Rate (per 100 person-years, 95% CI)	0	–	0.0 – 0.071	
Mean follow-up period (weeks, mean ± SD)	–	–	46.08 ± 7.80	

Safety outcomes

**Figure 1:**
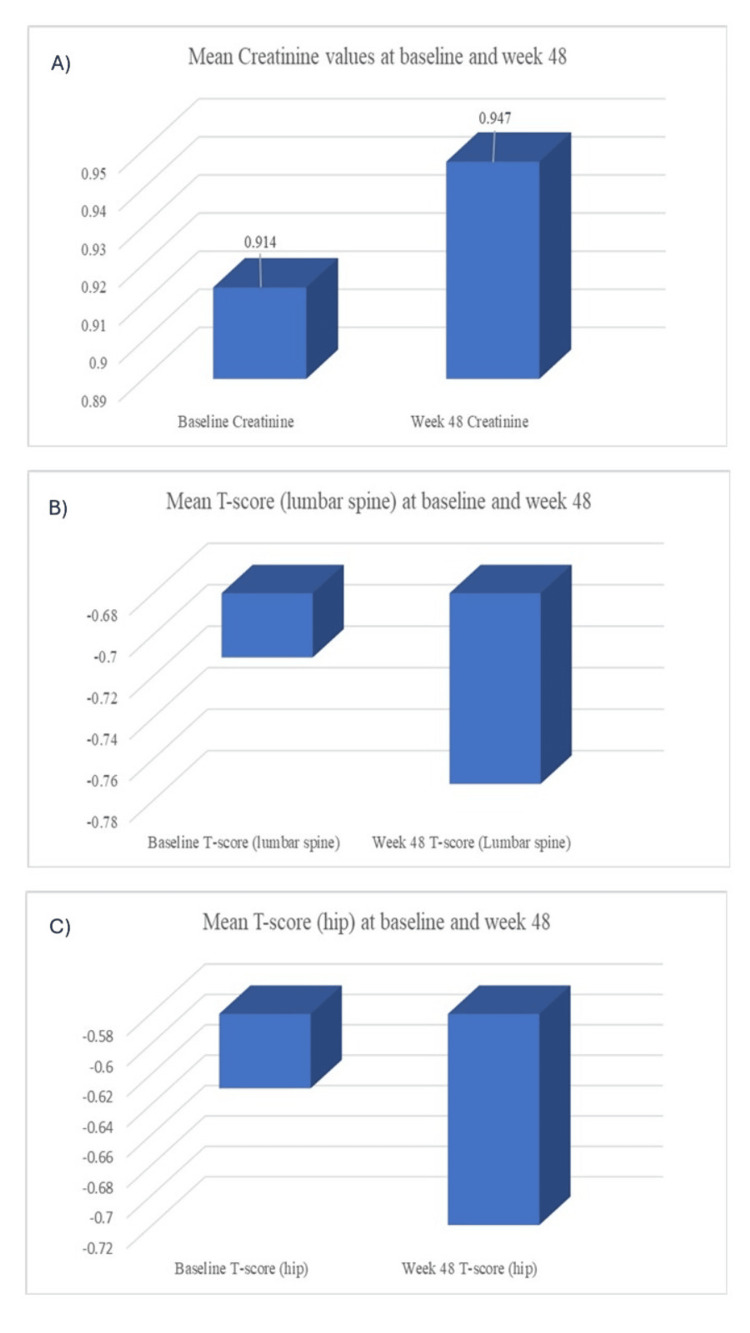
Renal function and bone mineral density from baseline to week 48: serum creatinine, lumbar spine T-score, and hip T-score. (A) Mean serum creatinine levels at baseline and week 48, expressed in mg/dL. (B) Mean T-score at the lumbar spine at baseline and week 48. (C) Mean T-score at the hip at baseline and week 48. Continuous variables are presented as mean values. Changes over time reflect within-group comparisons from baseline to week 48.

Adverse events were infrequent (6%, n=3) and mild. Two participants (4%) experienced mild GI symptoms. No moderate or severe AEs occurred, and no discontinuations were attributed to AEs. Renal safety monitoring showed a statistically significant but clinically minimal increase in mean serum creatinine from 0.914 ± 0.153 mg/dL at baseline to 0.947 ± 0.159 mg/dL at week (Figure 1A). The mean change was 0.0334 mg/dL, which was statistically significant but not clinically relevant. Moreover, no participant developed clinically significant renal impairment (eGFR <60 mL/min). Refer to Table 2 for more details.

**Table 2:** Safety Profile and Laboratory Monitoring Over 48 Weeks Renal, hepatic, and BMD parameters are summarized at baseline and week 48. eGFR decline and ALT elevations are reported based on clinically relevant thresholds. P-values reflect within-group comparisons over time; p < 0.05 denotes statistical significance. AE, Adverse events; ALT, Alanine transaminase; eGFR, Estimated glomerular filtration rate; GI, Gastrointestinal; HIV, Human Immunodeficiency Virus; ULN, Upper limit of normal; BMD, bone mineral density

Safety Parameter	n	Percentage (%)
AEs		
Any AEs	3	6.00
Mild GI symptoms	2	4.00
Moderate AEs	0	0.00
Severe AEs	0	0.00
Discontinuation due to AE	0	0.00
Renal Safety		
Baseline mean creatinine (mg/dL)	0.914 ± 0.153	
Week 48 mean creatinine (mg/dL)	0.947 ± 0.159	
Mean change from baseline	0.0334	
p-value	<0.0001	
Clinically significant renal impairment	0	0
eGFR decrease >25%	0	0
BMD (DEXA)		
Baseline mean T-score (lumbar spine)	-0.711± 1.171	
Week 48 mean T-score (lumbar spine)	-0.772± 1.157	
Mean change (%)	-8.58%	
p-value	0.017	
Baseline mean T-score (hip)	-0.629 ± 1.169	
Week 48 mean T-score (hip)	-0.719 ± 1.21	
Mean change (%)	-14.31%	
p-value	0.008	
Hepatic Safety		
Baseline mean ALT (U/L)	28.4 ± 8.43	
Week 48 mean ALT (U/L)	29.2 ± 9.04	
ALT elevation >2x ULN	0	0
Other Laboratory Abnormalities		
Hypophosphatemia	1	2.00
Proteinuria	0	0
Anaemia	0	0

Bone mineral density monitoring showed modest declines. Lumbar spine T-score decreased from -0.711 ± 1.171 to -0.772 ± 1.157 (p=0.017) (Figure 1B), while Hip T-score decreased from -0.629 ± 1.169 to -0.719 ± 1.21 (p=0.008) (Figure 1C). Although statistically significant, changes were small and remained within the normal range. Hepatic safety remained stable. Mean ALT level reported at baseline was 28.4 ± 8.43 U/L, and at week 48, ALT reported was 29.2 ± 9.04 U/L. No participant experienced ALT elevation >2 times the upper limit of normal. Other laboratory findings included one case of hypophosphatemia (2%) of the cohort. No cases of anemia, leukopenia, thrombocytopenia, or proteinuria were detected.

Adherence

**Figure 2:**
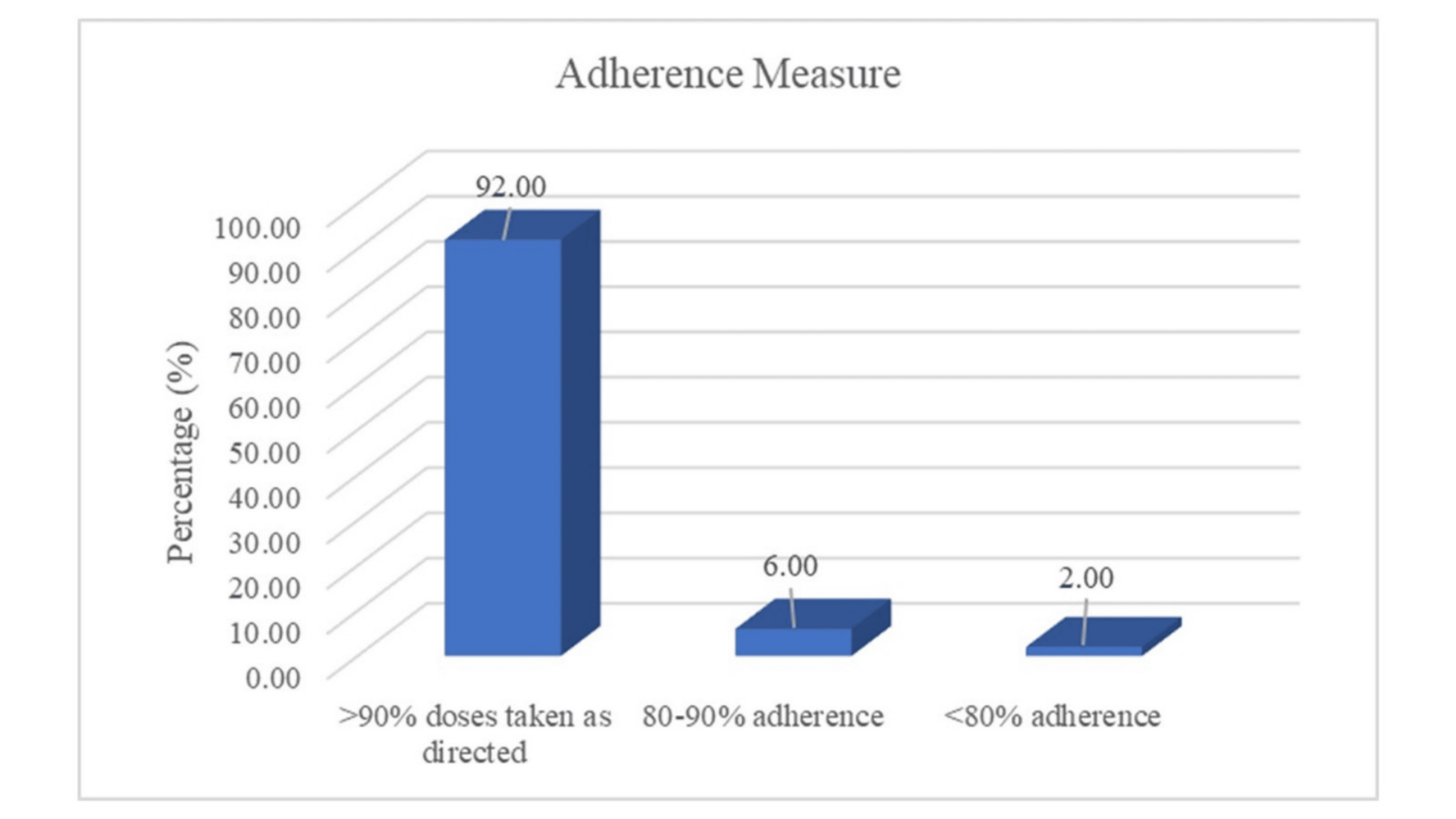
Self-reported adherence categories by regimen Bar chart showing the distribution of self-reported adherence categorized as >90%, 80-90%, and <80% of doses taken as directed. Values are expressed as percentages (%). Adherence categories are defined based on the proportion of prescribed doses taken during the study period.

Self-reported adherence was very high, with 92% of participants (46 individuals) indicating adherence exceeding 90%. Three participants (6%) reported adherence between 80% and 90%. One participant (2%) reported adherence below 80% (Figure 2). Daily PrEP users demonstrated 100% adherence. All 10 participants reported adherence greater than 90%. Daily PrEP users demonstrated 100% adherence, while on-demand PrEP users also showed strong adherence (92.5%). Thirty-seven participants (92.5%) reported adherence greater than 90%, while three participants (7.5%) reported adherence between 80% and 90%. Medication access challenges were noted. Ten participants (20%) experienced supply interruptions lasting 7-14 days. Nine participants (90%) of those affected had reserve pills to bridge the gap. All participants received adherence counseling. Refer to Table 3 for more details.

**Table 3:** Self-Reported Adherence and Regimen-Specific Adherence Overall adherence is categorized by the proportion of doses taken as directed (>90%, 80-90%, <80%). Regimen-specific adherence is stratified by daily and on-demand PrEP use. Medication supply interruptions are reported as median (range) in days. Adherence support interventions and barriers are described qualitatively. PrEP, Preexposure prophylaxis

Adherence Measure	n	Percentage (%)
Self-Reported Adherence		
Overall adherence rate	>90%	
>90% doses taken as directed	46	92.00
80-90% adherence	3	6.00
<80% adherence	1	2.00
Adherence by Regimen		
Daily PrEP total patient	10	
Daily PrEP - High adherence (>90%)	10	100
Daily PrEP - Moderate adherence (80-90%)	0	0
On-demand PrEP	40	
On-demand PrEP - High adherence (>90%)	37	92.50
On-demand PrEP - Moderate adherence (80-90%)	3	7.50
Barriers to Adherence		
Medication access issues	10	20.00
Adherence Support Interventions		
Received adherence counseling	50	100
Medication Supply Interruptions		
Experienced supply shortage	10	20.00
Duration of interruption (in days) (Range)	11 (7-14)	
Had reserve pills to bridge the gap	9	90.00

Sexually transmitted infections (STIs)

**Figure 3:**
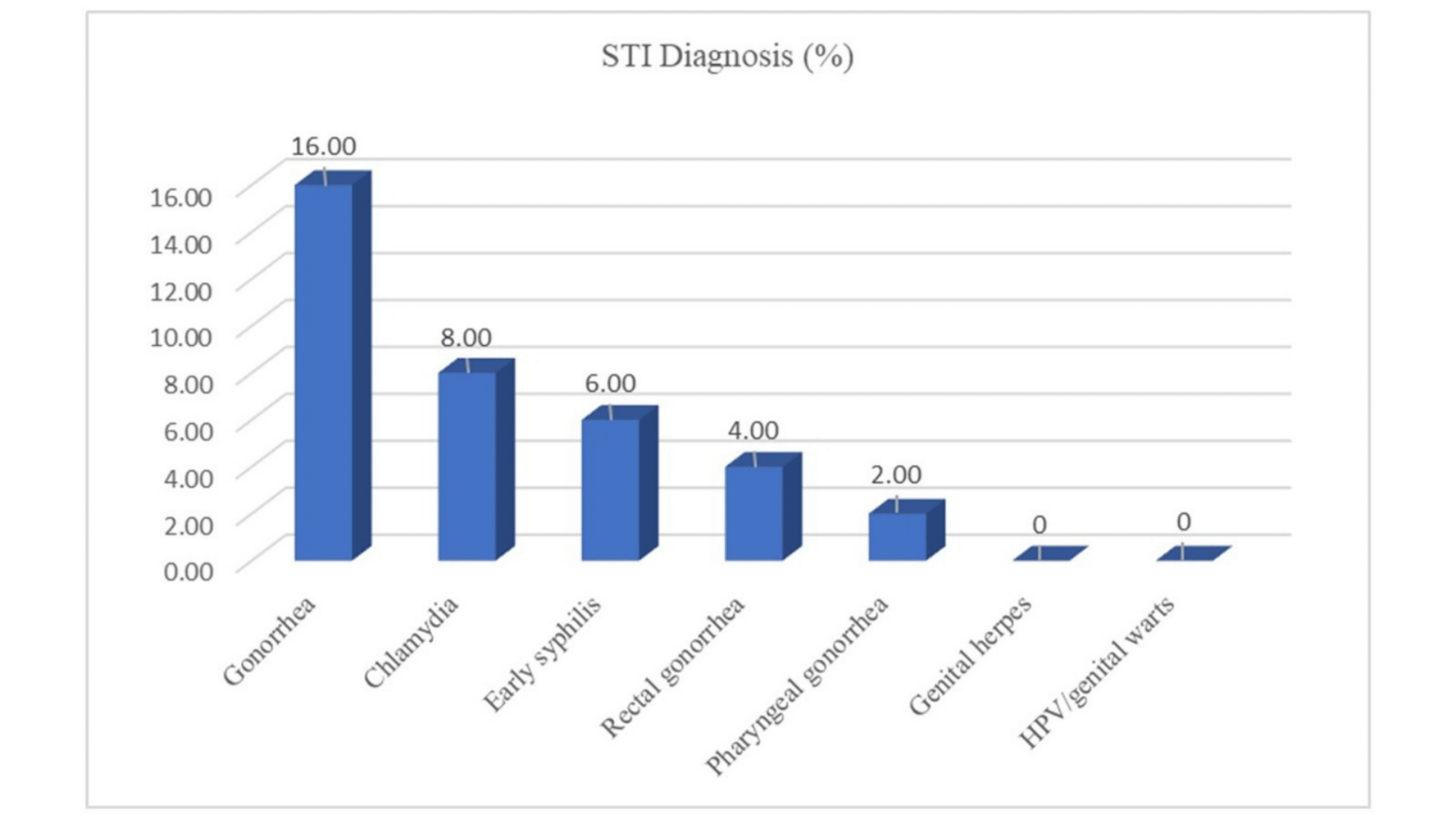
Distribution of STIs diagnosed during follow-up Bar chart illustrating the percentage (%) distribution of specific STI diagnoses during the study period, including gonorrhea, chlamydia, early syphilis, rectal gonorrhea, pharyngeal gonorrhea, etc. HPV, *Human papillomavirus*; STI, Sexually transmitted infection

Fifteen participants (30%) of the cohort were diagnosed with at least one new STI during follow-up. A total of 18 new STI infections were recorded, corresponding to an incidence rate of 35.4 per 100 person-years. The most frequent diagnoses were gonorrhea in eight participants (16%), chlamydia in four participants (8%), and early syphilis in three participants (6%). Rectal gonorrhea was detected in two participants (4%), and one case (2%) of pharyngeal gonorrhea was also reported. No cases of genital herpes or *Human papillomavirus *(HPV) were reported (Figure 3).

Repeat infections in participants with >1 STI occurred in three participants (20%). One participant was diagnosed with the same infection twice. All infections (100%) were treated promptly. Repeat testing was completed in all cases, and partner notification was performed in 15 cases (83.3%). Risk behavior remained high. Thirty-four cases, representing 68% of total participants, reported condomless sex. Twenty-three participants (46%) reported having multiple partners (>3 in the past 3 months). Refer to Table 4 for more details.

**Table 4:** Incidence of STI During Follow-Up STI incidence rates are expressed per 100 person-years. Overall incidence captures participants with ≥1 new STI and total diagnoses. Specific STIs are reported by type. STI by Risk Group, STI management, and Risk Behaviour Assessment are described qualitatively. HIV, Human Immunodeficiency Virus; STI, Sexually transmitted infection; HPV, *Human papillomavirus*

STI Type	n	Percentage (%)
Overall STI Incidence		
Participants with any new STI	15	30
Total new STI diagnoses	18	
STI incidence rate (per 100 person-years)	35.4	
Specific STI Diagnoses		
Gonorrhea	8	16.00
Chlamydia	4	8.00
Early syphilis	3	6.00
Rectal gonorrhea	2	4.00
Pharyngeal gonorrhea	1	2.00
Genital herpes	0	0
HPV/genital warts	0	0
STI by Risk Group		
Participants with HIV+ partners	4	26.67
Repeat STI Infections		
Participants with >1 STI during study	3	20.00
Participants with the same STI twice	1	6.67
STI Management		
All STIs treated promptly	18	100
Partner notification performed	15	83.3
Repeat testing after treatment	18	100
Risk Behavior Assessment		
Condomless sex reported	34	68.00
Multiple partners (>3 in 3 months)	23	46.00

Laboratory monitoring

The routine monitoring/laboratory tests confirmed safety and compliance. All participants underwent baseline HIV testing, and 245 follow-up HIV tests were performed, all of which were negative. Renal function test results showed no cases of eGFR <60 mL/min, and no proteinuria has been detected. Hepatitis screening identified two participants (4%) who were hepatitis B surface antigen (HBsAg)-positive at baseline. No new hepatitis infections have been reported. Complete blood count tests showed no cases of anemia, leukopenia, or thrombocytopenia. Refer to Table 5 for more details.

**Table 5:** Laboratory monitoring outcomes during PrEP follow-up Laboratory monitoring includes HIV testing, renal function monitoring, hepatitis screening, and complete blood count findings assessed during follow-up. HBsAg, Hepatitis B surface antigen; HCV, Hepatitis C virus; HIV, Human Immunodeficiency Virus; eGFR: estimated glomerular filtration rate

Laboratory Parameter	n	Percentage (%)
HIV Testing		
Baseline HIV tests performed	50	100
Follow-up HIV tests (total)	245	
Positive HIV tests	0	0
Indeterminate results requiring repeat	0	0
Renal Function Monitoring		
Baseline creatinine tests	50	100
Follow-up creatinine tests	245	
eGFR <60 at any timepoint	0	0
Proteinuria detected	0	0
Hepatitis Screening		
HBsAg positive at baseline	2	4.00
Anti-HCV positive at baseline	0	0
New hepatitis infections	0	0
Complete Blood Count		
Anaemia detected	0	0
Leukopenia detected	0	0
Thrombocytopenia detected	0	0

## Discussion

Our study provides real-world evidence from Saudi Arabia that PrEP with TAF/FTC is safe and effective in high-risk populations. The IPERGAY trial in France and Canada demonstrated that out of 199 participants from the TDF-FTC group, PrEP reduced HIV risk by 86% among high-risk MSM, supporting the effectiveness of PrEP [22]. Likewise, another study by Zivich et al used data from the iPrEx and DISCOVER trials to compare TAF/FTC with placebo. The study found that the risk of HIV infection among the participants (MSM) in the TAF/FTC group was 5.8% lower (95% CI: −2.0% to −9.6%) or 12.5-fold lower (95% CI: 0.02 to 0.31) than placebo, standardized to the DISCOVER population [23]. Further, another UK-based open-label randomized trial (PROUD) involved MSM and HIV negative gays (n=544) with PrEP implemented immediately (immediate group) or after a period of one year (deferred group). The study revealed that HIV incidence observed to be lower in immediate group (1.2 cases per 100 person-years, 90% CI 0.4-2.9) than deferred group (9.0 per 100 person-years, 90% CI 6.1-12.8; p=0.0001) with proportionate reduction of 86% (90% CI 64-96) and a rate difference of 7.8 per 100 person-years (90% CI 4.3-11.3) [24].

In our study, over the span of 48 weeks of follow-up, there were no observed HIV seroconversions among participants receiving PrEP, thereby underscoring its efficacy. Contrasting with our findings, another randomized, placebo-controlled trial conducted across South Africa, Uganda, and Zimbabwe, utilizing oral TDF, oral TDF-FTC, or 1% TFV vaginal gel in a cohort of 5,029 women, identified seroconversions in 334 participants [25].

Retention in care within our study was reported to be 92%, which is largely consistent with the outcomes of another randomized trial conducted by Baeten et al. In that study, participant retention remained at or above 96% throughout the duration, with 4,722 out of 4,747 individuals (99.5%) completing at least one HIV-1 test following randomization [11]. Another randomized, placebo-controlled trial conducted in South Africa, Uganda, and Zimbabwe with oral TDF, oral TDF-FTC, or 1% TFV vaginal gel with 5029 women reported that the rate of retention was 91% during 5509 person-years of follow-up [25].

High adherence is essential to achieve clinical benefits from antiretroviral agents for HIV-1 treatment. Adherence in our study was high, with 92% (n=46) of participants reporting intake of more than 90% of prescribed doses. This result was found to be consistent with another 96-week phase 3 trial conducted in Europe and North America, in which 5387 participants demonstrated high adherence to the study drug. In this study, approximately 78% to 82% of participants reported consuming the study drug for more than 95% of the time throughout all study visits. Median pill count adherence at week 96 was 98% (IQR 93-100%) [26]. Another trial conducted in 2120 women from Kenya, South Africa, and Tanzania with TDF-FTC or placebo reported that 95% of participants reported adherence to the assigned drug. Moreover, pill-count records indicated that participants took the study drug on 88% of the days it was available to them [27]. A systematic review and meta-analysis of 4129 records, including 43917 participants, reported a pooled suboptimal adherence of 37.7% within 6 months [28].

In our research, there were 18 new STI cases, including gonorrhea, chlamydia, and early syphilis, reported in 16%, 8%, and 6% of participants, respectively. Another study by Traeger et al., involving 1378 participants, also observed an increase in STI incidence from 69.5 to 98.4 cases per 100 person-years during follow-up (incidence rate ratio (IRR), 1.41 [95% CI, 1.29-1.56]) [29]. These findings align with a larger open-label extension study by Molina et al., where a high proportion of participants acquired at least one bacterial STI during follow-up, 43% (n=156). Specifically, 42% (n=113) had rectal infections, and overall, 23% (n=83) contracted chlamydia, 21% (n=76) gonorrhea, and 16% (n=57) syphilis [12].

In terms of safety monitoring, using PrEP showed a tolerable safety profile. Only mild GI symptoms in 4% of participants were reported, with no cases of discontinuations due to AEs and no clinically significant renal, hepatic, or bone toxicity. These findings contrast with results from a randomized trial of 400 HIV-negative participants, in which individuals receiving TDF-FTC experienced significantly higher rates of GI AEs (14% vs. 5%, P=0.002) and renal AEs (18% vs. 10%, P = 0.03) compared with placebo, despite similar rates of serious AEs across groups [22]. Similarly, this study reported that on‑demand PrEP use is linked to the same short‑term side effects observed with daily dosing, such as nausea, vomiting, diarrhea, abdominal discomfort, and other GI complaints [22, 30]. Another 72-week open-label extension (iPrEx OLE) trial, which enrolled MSM and transgender women (n=1603), experienced hypersensitivity (n=2) and other side effects (N=2, dizziness, nausea, and flatulence in one, weight gain in the other) [31].

Considering renal safety, no participant in our study developed clinically significant renal impairment (eGFR decline >25% or eGFR <60 mL/min). However, in contrast to our study, a recent data analysis from the ANRS‑IPERGAY trial and its open‑label extension study reported that, among 199 participants receiving TDF/FTC, baseline eGFR rate was <90 mL/min/1.73 m² in 26 individuals (13%) [32].

In our research, the lumbar spine T-score decreased from -0.711 ± 1.171 to -0.772 ± 1.157, (p=0.017) which is consistent with another study by Brown et al., where the participants in the oral TDF/FTC arm (n=117) experienced modest but measurable lumbar spine BMD declines of -93 (3.6%) at week 57 compared to long-acting cabotegravir (CAB-LA) arm 0.89% (3.1%) [33]. Similarly, a US based study by Havens et al., in HIV‑uninfected adolescent males on daily TDF/FTC PrEP reported that high drug exposure was associated with a significant decline in hip BMD (−1.59% vs +1.54% in low exposure, p = 0.001), supporting the observed reduction in hip T‑scores in our study participants (-0.629 ± 1.169 to -0.719 ± 1.21, (p=0.008)) [34]. These findings underscore the importance of regimen selection for individuals at risk of osteoporosis or fracture.

The strengths of this study encompass meticulous monitoring of renal, hepatic, and bone safety parameters, high retention and adherence rates, and the demonstration of feasibility within a conservative sociocultural context. However, this study's limitations include a modest sample size and a single-center design. Future research should therefore focus on larger, multi-center cohorts, extended safety monitoring over longer periods, and strategies for integrating PrEP within treatment regimens.

## Conclusions

This real-world, single-center study demonstrates that tenofovir alafenamide/emtricitabine-based PrEP is highly effective, safe, and practical for HIV prevention among high-risk individuals in Saudi Arabia, with no seroconversions, high retention, and excellent adherence observed over 48 weeks. The favorable safety profile, coupled with strong adherence across both daily and on-demand regimens, reinforces the practicality of PrEP implementation in routine clinical settings. However, the notable incidence of STIs and persistently high-risk behaviors highlights the need for integrated prevention strategies, including regular screening, behavioral interventions, and adherence support. Overall, these findings provide important region-specific evidence to inform policy and support the scale-up of PrEP programs in the MENA region, while emphasizing the need for larger, multi-center studies to further validate long-term outcomes.
